# Whole-body cryostimulation as an effective method of reducing low-grade inflammation in obese men

**DOI:** 10.1007/s12576-013-0269-4

**Published:** 2013-06-07

**Authors:** Ewa Ziemann, Robert A. Olek, Tomasz Grzywacz, Jędrzej Antosiewicz, Sylwester Kujach, Marcin Łuszczyk, Mirosław Smaruj, Ewelina Śledziewska, Radosław Laskowski

**Affiliations:** 1 Department of Physiology, Gdansk University of Physical Education and Sport, Kazimierza Górskiego 1, 80-336 Gdansk, Poland; 2 Department of Biochemistry, Gdansk University of Physical Education and Sport, Kazimierza Górskiego 1, 80-336 Gdansk, Poland; 3 Department of Sport Psychology, Warsaw School of Social Sciences and Humanities, Warsaw, Chodakowska 19/31, 03-815 Warszawa, Poland; 4 Department of the Theory of Sport, Gdansk University of Physical Education and Sport, Kazimierza Górskiego 1, 80-336 Gdansk, Poland

**Keywords:** Adipocytokines, Cold exposure, Visceral fat tissue, Muscle mass

## Abstract

This study was aimed to evaluate anti-inflammatory effect of the whole body cryostimulation in obese men. Fourteen subjects (BMI >30 kg m^−2^), divided into two subgroups according to cardiorespiratory fitness: higher (HCF) or lower (LCF), have been exposed to 10 sessions in a cryogenic chamber (−110 °C). Blood samples were collected before, 30 min and 24 h after the first, fifth and last cryostimulation. Coldness exposures affected blood cytokine profile; however, the response depended on subjects’ fitness capacity. Concentrations of pro-inflammatory cytokines in the LCF decreased by 19, 6.8, and 7.4 % in IL-6, resistin, and visfatin, respectively. TNFα in the LCF dropped 4.3-fold compared to baseline, while in the HCF, changes were smaller, yet significant. Anti-inflammatory cytokine IL-10 increased in both groups. No changes in adiponectin and leptin were observed in either group. Obtained results suggest that whole body cryostimulation can be a supplementary method for obese in reducing systemic inflammation.

## Introduction

Recent reports on obesity have shown an increasing prevalence of this condition since the 1990s, which is also predicted to grow even further in European populations by 2015 [1]. Adipose tissue is the important, regulatory factor of many pathological processes that accompany obesity [2]. Certain cytokines produced by this tissue are thought to provide an important link between obesity, insulin resistance, and related inflammatory disorders [3, 4]. Moreover, in obese individuals, adipose tissue contains a large number of macrophages, which are the source of TNFα, IL-6 and other cytokines [5]. Circulating cytokines regulate energy homeostasis, neuroendocrine, and immune functions; however, the presence of an excessive amount of fat tissue and resulting, increased level of some pro-inflammatory cytokines have been observed to lead to low-grade systemic inflammation in obese individuals [2, 6, 7].

Physical exercise is one of the mechanisms that may be responsible for the anti-inflammatory response and protection against chronic medical disorders associated with low-grade inflammation [8]. During exercise, skeletal muscles produce and release myokines, which may induce the anti-inflammatory effect and act as energy sensors, exerting both local and endocrine metabolic agents [9]. Furthermore, some of these myokines might change the metabolism of white fat tissue [10]. Data on physical exercise characteristics (type, duration, and intensity) ensuring an effective reduction in inflammation markers have not yet been obtained [11, 12]. Aerobic exercise combined with resistance exercise has been shown to have a greater anti-inflammatory effect than each separately [13]. Nonetheless, some studies have presented inconsistent findings regarding the anti-inflammatory effect of exercise [14]. In several studies, exercise did not trigger any declines in the levels of pro-inflammatory cytokines TNFα, IL-1β, and CRP [14–16]. In addition to exercise, diet and medically-induced weight loss have been previously shown to attenuate inflammation [17, 18] and to promote lipid utilization in skeletal muscle in obese people [19]. Regardless, some studies have reported that these treatments cause weight loss only, without reducing the inflammatory state. For example, CRP and IL-6 were shown to drop in morbidly obese patients as a result of a bariatric surgery; however, changes in TNFα were not equivocal [20]. Furthermore, a 25 % calorie restriction in diet in conjunction with an aerobic training-induced weight loss did not impact markers of systemic inflammation or expression of inflammation-related adipose genes in overweight individuals [21]. Overall, high demand for effective, complementary methods for reducing systemic low-grade inflammation is highly justified.

Whole-body cryostimulation may be an alternative method for reducing inflammation [22–24]. It has been shown to reduce pro-inflammatory response, relieve pain more effectively than other local cryotherapies and enhance muscles’ post-exercise recovery [25, 26]. What is more, whole-body cryostimulation caused changes in the cytokine profile in healthy men. Twenty sessions of this treatment resulted in an increase of the anti-inflammatory cytokine IL-10 and pleiotropic cytokine IL-6 and a decrease of pro-inflammatory IL-1α [27]. Additionally, other cold treatments induced changes in the cytokine levels of adipose tissue, adiponectin, resistin, and leptin [28, 29]; however, the applied temperatures were far from the extreme temperature used in whole-body cryostimulation.

To our knowledge, no studies have so far investigated the effect of whole-body cryostimulation on the secretion of hormones and cytokines in obese individuals. Thus, the present study was designed to evaluate the effects of whole-body cryostimulation in two stages. First, we determined the influence of physical cardiorespiratory fitness capacity on the concentration of adipocytokines in obese men. Second, we assessed the effects of whole-body cryostimulation on adipocytokines in two groups of obese men characterised by either higher (HCF) or lower fitness capacity (LCF). Basing on previous investigations, our primary hypothesis assumed that whole-body cryostimulation would reduce low-grade systemic inflammation in obese men and induce significant growth in the anti-inflammatory cytokine level. Finally, we verified whether and how the immunological response depended on subjects’ cardiorespiratory capacity fitness.

## Methods

### Subjects

Fourteen obese men participated in the experiment (40 ± 4.0 years of age, height of 178 ± 3.0 cm, HCF weight of 99.8 ± 4.6 kg, and LCF weight of 107.5 ± 12.1 kg). They were recruited via an advertisement placed in a local newspaper. All the subjects underwent a medical check-up before the exercise test and being submitted to cold exposure. Those with uncontrolled hypertension (diastolic blood pressure over 100 mm Hg), a history of cardiac arrhythmia, cardio-respiratory disorders, and cold allergy were excluded from the study. The participants were categorised as obese based on their BMI according to the current guidelines (BMI >30 kg m^−2^) [30]. All patients were medication-free. The participants had never previously been subjected to any form of cryotherapy. Written, informed consent was obtained from all subjects. All procedures were approved by the Bioethical Committee of the Regional Medical Society in Gdansk NKEBN/245/2009 and conformed to the standards set by the Declaration of Helsinki.

**Fig. 1:**
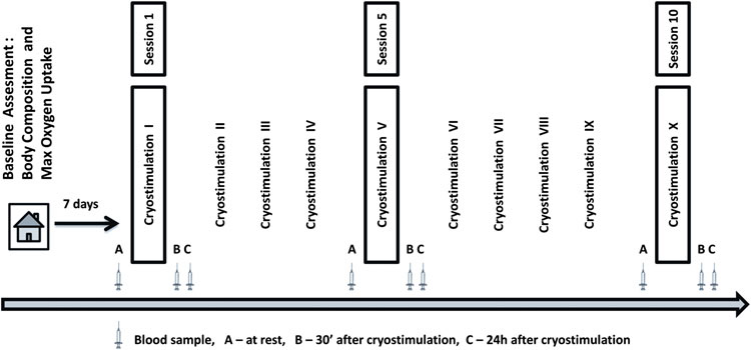
The study design

One week prior to the start of the experiment, body composition and aerobic capacity were determined for each participant. The group was divided into two subgroups according to the participants’ cardiorespiratory fitness, which had been measured in terms of the maximal oxygen consumption (VO_2max_). Based on the classification proposed by Astrand [31], subjects with VO_2max_ above 35 mL kg^−1^ min^−1^ were assigned to the HCF group, whereas those with the lower VO_2max_ were enrolled in the LCF group. The HCF group (via a verbal survey) described its regular, long-term physical activity as 3–4 h per week of aerobic exercise with moderate intensity, including some resistance drills. During the experiment, all participants were instructed not to change any aspect of their daily habits regarding, e.g., diet. However, to explicitly assess the effect of cryostimulation on cytokine concentration, subjects were asked to avoid any form of exercise or other medical treatment. The schedule of the experiment is presented in Fig. 1.

### Body composition assessment

Body mass (BM) and body composition were estimated using a multi-frequency impedance plethysmograph body composition analyser (In Body 720; Biospace Analyzer, Korea). This analyser accurately measures the amount of body water and body composition, including fat mass, free fat mass, skeletal muscle mass and soft lean mass. Additionally, the visceral fat area was determined and expressed in cm^2^. Precision of the repeated measurements was expressed as the coefficient of variation, which on average was 0.6 % for fat mass percentage [32]. The measurements were taken 1 h before breakfast. The participants had emptied their bladders and bowels prior to the assessment. During the measurement, the participants wore only briefs and remained barefoot. The body composition analysis was repeated 1 day after the procedure of 10 sessions of whole body cryostimulation.

### Cardiorespiratory fitness measurement

To determine VO_2max_, the participants performed a graded cycle ergometry test on an electromagnetically braked, cycle ergometer (ER 900; Jaeger/Viasys Health Care, Germany). The participants were allowed a 5-min warm up at an intensity of 1.5 W kg^−1^, with a pedalling cadence of 60 rpm. Immediately following the warm-up, the participants began VO_2max_ testing by cycling at a gradually increasing workload by 25 W min^−1^ until the participant reached the point of volitional exhaustion [33, 34]. The recovery was passive, with the participant in a seated position. Breath-by-breath pulmonary gas exchange was measured throughout the VO_2max_ test; the O_2_ and CO_2_ analysers were calibrated prior to each test using standard gases of known concentrations in accordance with manufacturer guidelines.

### Whole-body cryostimulation

All the participants were subjected to a series of coldness exposures (once a day at 0930 hours, 10 sessions per series) in a cryogenic chamber at the Pomeranian Rheumatologic Centre in Sopot, Poland, conducted by highly qualified, medical staff. Each cryostimulation session lasted 3 min at a temperature of −110 °C. Entry into the cryo-chamber was preceded by a 20- to 30-s adaptation in the vestibule at a temperature of −60 °C. The subjects were dressed in shorts, socks, gloves, and a hat covering their auricles. Each exposure was preceded by a light breakfast between 0700 and 0730 hours according to the instructions given to the subjects.

### Blood analysis

Blood samples were collected at 3 stages of the experiment: before and 24 h after the 1st, 5th and 10th coldness exposure. Additionally, based on data from a published study and changes in adipokines concentration after cold exposure [28], blood was collected 30 min after the selected exposures (Fig. 1). Blood samples were taken from the antecubital vein into the vacutainer tubes with EDTAK_2_. Immediately after collection, the samples were divided into two portions. One portion was used for haematological measurements determined by conventional methods using a COULTER^®^ LH 750 Hematology Analyzer (Beckman-Coulter, USA). The other portion was transferred to centrifuge tubes containing aprotinin (catalogue no. RK-APRO) from Phoenix Pharmaceuticals. The final concentration of aprotinin was 0.6 Trypsin Inhibitor Unit/1 mL of blood. The samples were centrifuged at 2,000*g* for 10 min at 4 °C. The separated plasma samples were frozen and kept at −70 °C until later analysis. Plasma interleukins: IL-6, IL-10, and tumour necrosis factor alpha, TNFα, were determined by enzyme immunoassay methods using commercial kits (catalogue nos. HS600B, HS100C, HSTA00D; R&D Systems, USA). An enzyme immunoassay method using commercially available kits from Phoenix Pharmaceuticals. was employed to determine the plasma visfatin C-terminus (catalogue no. EK-003-80) and adiponectin (catalogue no. EK-ADI-01), and kits from R&D Systems were used to determine the levels of leptin (catalogue no. DLP00), leptin-soluble receptor (catalogue no. DOBR00) and resistin (catalogue no. DRSN00).

### Statistical calculations

Statistical analysis was performed using Statistica 8.0 (StatSoft, USA) for Windows. A 2 (group) × 2 (time) repeated measures analysis of variance (ANOVA) was used to determine the differences between the groups in the pre-, during and post-cryostimulation sessions. The level of significance was set at 0.05 for all analyses. Additionally, to elaborate on the differential significance between groups before and after whole-body cryostimulation, a method of multiple comparison (post hoc–HSD Tukey’s) was applied. Statistical calculation presented in Table 1 were performed basing on data from all points of blood collection. Values of *p* based on all blood collection samples are presented in Table 1. In the “Results” section, *p* values refer to statistical differences of measurements for particular blood collections (post hoc). Furthermore, due to the small number of subjects, we also calculated the effect size (partial η^2^) by ANOVA, ranging between 0 and 1. Using Cohen’s rule of thumb as well as the conversion table for eta squared, the interpretations of the partial η^2^ value are unequivocal. However, the most restrictive interpretation method assigns values of partial η^2^ to the effect size as follows: 0.1 constituted a small effect, 0.3 represented a medium effect, and values above 0.5 represented a large effect.

**Table 1:** The effect of 10 sessions of whole-body cryostimulation on immunological response and adipokines concentration in obese men

Variable	Before I cryo		Before V cryo		24 h after V cryo		24 h after X cryo		Differences	*P* value	Effect size
	HCF	LCF	HCF	LCF	HCF	LCF	HCF	LCF			
IL-6 (pg mL^−1^)	1.3 ± 0.2	2.1 ± 0.9	1.0 ± 0.2	2.0 ± 1.0	1.8 ± 0.5	2.1 ± 0.7	1.2 ± 0.2	1.7 ± 0.7	Time	0.01	0.18
									Group	0.02	0.36
TNF α (pg mL^−1^)	1.3 ± 0.4	1.3 ± 1.3	0.9 ± 0.3	0.7 ± 0.4	0.9 ± 0.2	0.4 ± 0.1*	1.0 ± 0.2	0.3 ± 0.1*	Time	0.002	0.23
									Group	0.002	0.59
									Time × group	0.0004	0.27
IL-10 (pg mL^−1^)	3.9 ± 0.1	4.5 ± 0.8	3.8 ± 0.7	5.0 ± 0.7	4.7 ± 0.1	5.2 ± 0.8	4.3 ± 0.2	5.5 ± 1.0	Time	0.000001	0.48
									Group	0.005	0.49
									Time × group	0.003	0.21
Leptin (ng mL^−1^)	5.1 ± 0.4	36.8 ± 19.3*	5.9 ± 0.3	35.8 ± 20.4*	7.9 ± 1.8	31.3 ± 17.1*	6.2 ± 0.8	32.6 ± 14.5*	Group	0.001	0.59
									Time × group	0.001	0.22
Leptin sR (ng mL^−1^)	30.9 ± 2.9	22.5 ± 4.0*	31.1 ± 2.0	21.1 ± 2.9*	29.1 ± 0.6	20.5 ± 3.0*	28.1 ± 2.6	21.3 ± 5.7*	Time	0.01	0.17
									Group	0.0001	0.75
Adiponectin (ng mL^−1^)	1,751.2 ± 266.2	1,292.5 ± 557.5	1,796.8 ± 341.8	1,163.9 ± 719.0	1,581.2 ± 206.6	1,296.8 ± 447.1	1,801.0 ± 215.9	1,290.2 ± 410.5	Group	0.05	0.28
Resistin (ng mL^−1^)	9.6 ± 1.1	17.6 ± 5.2*	10.2 ± 1.5	16.4 ± 4.2	11.9 ± 0.5	15.0 ± 4.2	10.5 ± 0.6	16.4 ± 6.4	Time	0.000005	0.33
									Group	0.01	0.42
									Time × group	0.007	0.19
Visfatin (ng mL^−1^)	13.1 ± 0.6	16.1 ± 3.1	13.6 ± 2.2	13.8 ± 1.3	13.7 ± 1.1	14.5 ± 1.7	16.2 ± 0.5	14.9 ± 2.4	Time	0.002	0.23
									Time × group	0.002	0.24
Visfatin/VFA (ng mL^−1^ cm^−2^)	0.110 ± 0.006	0.094 ± 0.022	0.113 ± 0.016	0.081 ± 0.012*	0.114 ± 0.001	0.085 ± 0.015*	0.137 ± 0.013	0.086 ± 0.015*	Time	0.001	0.25
									Group	0.0001	0.78
									Time × group	0.0004	0.27

Values are mean ± SD

*IL-6* interleukin-6, *TNF α* tumor necrosis factor alpha, *VFA* visceral fat area, effect size expressed as partial η^2^, *HCF* higher cardiorespiratory fitness, *LCF* lower cardiorespiratory fitness, *Cryo* cryostimulation, *24 h* after cryostimulation, power test was in range 0.77–1.0, differences: *time* comparison before and after cryostimulation, *group* differences between HCF and LCF, *time* × *group* interactions between group and time

* Significantly different from HCF group: *p* < 0.05 for post hoc analysis

## Results

All participants completed the study, with no adverse events being reported.

### Baseline data

The basic anthropometric and physiological characteristics of the subjects are summarised in Table 2. All participants were categorised as obese, based on the average BMI of the HCF (31.4 ± 2.0 kg m^−2^) and LCF group (34.0 ± 2.5 kg m^−2^). The absolute fat mass and percentage of fat tissue values exceeded the recommended ranges for subject age. Additionally, in both groups, an elevated amount of visceral fat area (VFA) was observed (Table 2). However, compared to the LCF group, the HCF group was characterised by significantly lower fat content, higher free fat mass (FFM) and skeletal muscle mass (SMM). All differences were statistically significant (Table 2).

**Table 2:** Anthropometric and physiological characteristics of participants

Variable	HCF group	LCF group	*P* value
FFM (kg)	76.5 ± 2.7	68.6 ± 5.4	0.004
SMM (kg)	43.8 ± 1.6	39.0 ± 3.4	0.005
Fat (kg)	23.2 ± 3.2	38.9 ± 8.2	0.0007
Fat (%)	23.2 ± 2.5	35.8 ± 4.2	0.0002
VFA (cm^2^)	122.6 ± 9.5	173.7 ± 30.5	0.001
VO_2max_ (mL kg^−1^ min^−1^)	43.0 ± 3.0	25.7 ± 2.0	0.0002
VO_2max_ (mL ${\text{kg}}_{\text{SMM}}^{ - 1}$ min^−1^)	99.9 ± 4.8	71.1 ± 9.6	0.00004

Values are mean ± SD

*FFM* free fat mass, *SMM* skeletal muscle mass, *Fat* fat mass, *Fat*  *%* percentage of body fat, *VFA* visceral fat area, *VO*_*2max*_ maximal oxygen uptake expressed in relatively values and per kg skeletal muscle mass, *HCF *(*n* = 7) higher cardiorespiratory fitness, *LCF *(*n* = 7) lower cardiorespiratory fitness

The average values of maximal oxygen uptake were lower in the LCF group than the HCF group (*p* < 0.001). The discrepancy between groups advanced when the relative maximal oxygen uptake was calculated per kg of SMM, confirming that the HCF group had higher cardiorespiratory fitness than the LCF group (Table 2).

**Table 3:** Hematological parameters of subjects at the baseline and 24 h after the last session of cryostimulation

Variable	Before cryostimulation		24 h after last cryostimulation		*P* value at baseline	*P* value 24 h after XC
	HCF group	LCF group	HCF group	LCF group		
White blood cells (10^3^ μL)	5.9 ± 1.1	7.6 ± 1.0	6.5 ± 1.5	7.5 ± 0.5	0.01	0.01
Neutrophiles (%)	55.2 ± 10.4	49.4 ± 6.7	47.0 ± 7.1	48.9 ± 7.2	ns	ns
Lymphocytes (%)	31.0 ± 8.9	39.7 ± 6.1	40.0 ± 6.5	39.8 ± 6.7	ns	ns
Monocytes (%)	10.0 ± 1.9	7.5 ± 0.7	8.0 ± 0.7	7.5 ± 0.5	0.007	ns
Eosinophiles (%)	3.2 ± 1.0	2.4 ± 0.8	3.0 ± 1.5	2.5 ± 0.8	ns	ns
Basophiles (%)	0.5 ± 0.3	1.1 ± 0.3	1.1 ± 0.3	1.1 ± 0.2	0.004	ns
Red blood cells (10^6^ μL)	5.0 ± 0.1	5.4 ± 0.3	5.1 ± 0.3	5.4 ± 0.4	0.007	0.05
Hemoglobin (g dL^−1^)	15.2 ± 0.1	15.9 ± 0.9	15.4 ± 1.1	15.8 ± 0.9	ns	ns
Hematocrit (%)	43.6 ± 0.8	47.1 ± 2.2	44.0 ± 2.0	47.3 ± 2.3	0.002	0.01
Thrombocytes (10^3^ μL)	209.0 ± 17.5	273.5 ± 29.2	259.0 ± 35.5	271.1 ± 31.5	0.0005	ns

Values are mean ± SD

*ns* no significant statistical differences between groups, *HCF* higher cardiorespiratory fitness, *LCF* lower cardiorespiratory fitness, *XC* last (10) whole body cryostimulation

**Fig. 2:**
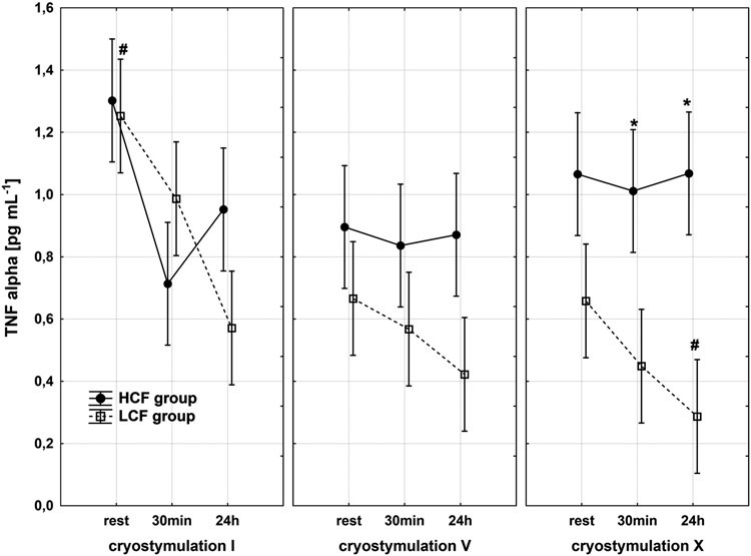
Changes in TNFα concentration in HCF and LCF group during the whole exposure period, *significantly different from HCF group: *p* < 0.05 for post hoc analysis

Haematological parameter analysis revealed that all values were within the reference range; however, the LCF group displayed higher amounts of red blood cells (*p* < 0.01) and thrombocytes (*p* < 0.001) compared with the HCF group. Also, a discrepancy between groups in white blood cells was observed (Table 3). To estimate if our obese subjects experienced low-grade systemic inflammation, pro- and anti-inflammatory cytokines were measured. The immunological data showed that, compared with the LCF, the HCF group had lower concentrations of IL-6 (*p* < 0.05) and IL-10 (*p* < 0.05). Both groups presented elevated concentrations of the pro-inflammatory cytokine TNFα, yet the ranges of groups’ values indicated major diversification (Table 1). For the HCF group, the concentration of TNFα ranged from min 0.6 pg mL^−1^ to max 1.5 pg mL^−1^, whereas the LCF group varied from min 0.4 pg mL^−1^ to max. 4.0 pg mL^−1^ (Fig 2). In both groups, a significant, inverse correlation between TNFα and VO_2max_ was observed. The higher the maximal oxygen uptake, the lowest was the pro-inflammatory TNFα concentration (for HCF group *r* = −77 and for LCF *r* = −0.75, *p* < 0.05).

Differences between groups were also recorded in concentrations of fat tissue hormones (adipokines). Subjects in the HCF group were characterised by lower concentrations of leptin (*p* < 0.001) and higher concentrations of leptin receptor (leptin sR) (*p* < 0.001) than the LCF group. Concentrations of the other pro-inflammatory adipokines, resistin and visfatin, were also lower in the HCF group (*p* < 0.002 and *p* < 0.05, respectively) (Table 1). Regardless of an unclear role of visfatin in inflammation and discrepancies in the interpretation of its concentrations in relation to body mass components [2], visfatin values per cm^2^ visceral fat area were calculated, revealing that significant differences between groups were also present (Table 1).

Furthermore, significant differences in the anti-inflammatory adipokine adiponectin between groups were observed. The HCF group was characterised by a higher concentration compared with the LCF group. Differences between groups were statistically significant and post hoc analysis revealed *p* < 0.05 for the baseline values.

In the first stage of the experiment (prior to applying cryostimulation procedure), we aimed to investigate whether the cardiorespiratory fitness and muscle mass affected adipokines concentration in obese men. Overall, greater values of VO_2max_ and muscle mass revealed lower resistin and higher leptin receptor concentration for all subjects (Table 4). Moreover, when the maximal oxygen uptake was calculated per skeletal muscle mass, it correlated with the concentrations of adipokines: negatively with leptin and positively with leptin sR (Table 4).

**Table 4:** The influence of cardiorespiratory fitness and body composition on adipokines concentration among all participants before and after the cryostimulation

Variable	Leptin (pg mL^−1^)		Leptin sR (ng mL^−1^)		Adiponectin (ng mL^−1^)		Resistin (ng mL^−1^)		Visfatin (ng mL^−1^)	
	BC	24 h AC	BC	24 h AC	BC	24 h AC	BC	24 h AC	BC	24 h AC
SMM (kg)	−0.30	−0.34	0.69*	0.62*	0.52	0.70*	−0.91*	−0.90*	−0.11	0.54*
Fat (kg)	0.66*	0.71*	−0.60*	−0.63*	−0.28	−0.31	0.44	0.33	0.60*	0.10
Fat (%)	0.65*	0.70*	−0.70*	−0.67*	−0.40	−0.48	0.66*	0.55*	0.54*	−0.28
VFA (cm^2^)	0.64*	0.69*	−0.49	−0.48	−0.24	0.20	0.37	−0.30	0.60*	−0.10
VO_2max_ (mL kg^−1^ min^−1^)	−0.80*	0.81*	0.70*	0.55	0.35	0.52	−0.67*	−0.48	−0.60*	0.40
VO_2max_ (mL ${\text{kg}}_{\text{SMM}}^{ - 1}$ min^−1^)	−0.77*	−0.70	0.70*	0.33	0.32	0.48	−0.61*	−0.33	−0.55	0.15

Values are Pearson correlation

*BC* before cryostimulation, *24* *h AC* after last cryostimulation, *SMM* skeletal muscle mass, *Fat* fat mass, *Fat* *%* percentage of body fat, *VFA* visceral fat area, *VO*_*2max*_ maximal oxygen uptake

* Values were significant at *p* < 0.05

### Effect of 10 sessions of whole-body cryostimulation

Immunological response and changes in adipokines concentrations after the whole body cryostimulation are presented in Table 1. According to our expectations, exposure to coldness did not cause any changes in body composition of our subjects (data not shown). Moreover, this treatment did not modify haematological parameters; thus, most initially observed differences between groups were maintained (Table 3). Nevertheless, 10 sessions of whole-body cryostimulation induced immunological alterations. The ranges and tendencies of these changes were differentiated in both groups (Table 1). Values recorded in the LCF group 24 h after the last coldness exposure triggered decreases in the concentrations of IL-6 and TNFα compared to the baseline. This descending trend of TNFα was maintained in the LCF group throughout the whole period of cryostimulation, with the trend being the most dynamic during the first 5 days of the series (Fig. 2). The drop in TNFα concentration in the LCF group, recorded 24 h after the 5th session, was statistically significant compared to the baseline. The subsequent sessions of cryostimulation series enhanced this decrease. Following the coldness treatment, a decrease in TNFα was also observed in the HCF group, yet the drop was less pronounced (1.3 at baseline to 1.0 pg mL^−1^ 24 h after the last cryostimulation). At the same time, the concentration of IL-6 remained unchanged (Table 1).

Simultaneously, significant elevation of the anti-inflammatory cytokine IL-10 was noted in both groups (*p* < 0.05 in HCF and *p* < 0.001 in LCF for values before and 24 h after the 10th session). The rise of IL-10 maintained in LCF group was maintained after the 5th and sustained 24 h after the last sessions. Repeated measures analysis of variance including all blood collection points indicated no significant differences (Table 1).

Furthermore, alternations in the concentrations of adipokines during and after whole-body cryostimulation were investigated. The concentration of leptin and leptin receptor did not change significantly in either group (Table 1). Moreover, exposures series did not trigger the elevation of adiponectin in either group. Nonetheless, positive correlations between SMM and adiponectin concentration recorded at baseline among all participants, reached statistical significance after the 10th session of cryostimulation (Table 4).

Variations of resistin and visfatin concentrations caused by cryostimulation followed divergent trends in both groups (Table 1). However, in both groups, changes resulted from the applied coldness exposures, as suggested by the effect size for group-time interaction of 19 and 21 % for resistin and visfatin, respectively. After 10 sessions of whole-body cryostimulation, concentrations of resistin and visfatin in the HCF group increased compared to baseline by 9.4 and 23.4 %, respectively. Conversely, in the LCF group, the resistin and visfatin levels subsequently dropped by 6.8 and 7.4 %, respectively.

Unexpectedly, 24 h after the 10th session of cryostimulation, subjects characterised by higher skeletal muscle mass showed significantly higher concentrations of visfatin. The correlation between the maximal oxygen uptake and adipokines, observed at baseline in all participants, was maintained after the complete series of coldness exposures (Table 4).

## Discussion

In the present study, we were able to demonstrate that whole-body cryostimulation is effective in reducing low-grade inflammation. The obtained data revealed that 10 sessions of cryostimulation caused a significant decrease in TNFα concentration. At baseline, subjects exhibited elevated concentrations of TNFα, very likely due to high levels of fat tissue, especially in the visceral fat area. This suggests that participants may have been experiencing low-grade systemic inflammation, which is consistent with previously presented results [35]. Decreasing, post-cryostimulation TNFα levels observed 24 h after the last exposure corresponded with the Hirvonen et al. [22] findings, who observed that 7-day whole-body cryostimulation effectively reduced the inflammation process in rheumatoid arthritis patients. Additionally, our study showed that cryostimulation-induced changes in TNFα concentrations were affected by cardiorespiratory fitness. In the LCF group, whole-body cryostimulation caused a 4.3-fold decrease in TNFα levels, whereas in the HCF group, the recorded drop was smaller, yet still significant. Although the anti-inflammatory effect of exercise has been well documented [8, 36], the HCF group’s results strongly suggest that cryostimulation might have potentiated this impact of physical activity. At the same time, in the HCF group, the whole body cryostimulation enhanced the inversely correlation between TNFα and cardiorespiratory fitness, which was noted at baseline. In our previous published study, this procedure has been shown to effectively reduce TNFα concentrations in overreached athletes [37]. Decreased values of TNFα may be particularly significant in obese individuals, not only with respect to an inflammatory state reduction but also in regulating and interfering with energy metabolism, especially lipid homeostasis [38].

Whole-body cryostimulation increases IL-6 concentrations in highly trained tennis players as well as in untrained non-obese men [23, 37]. In contrast, in the present study, IL-6 decreased in the LCF group, yet in the HCF remained almost constant. It is worth noting that IL-6 values at baseline varied between groups; the concentration in the HCF group was lower than in the LCF group. Such results may be attributed to the higher level of physical activity demonstrated by the HCF subjects, which has been reported to reduce IL-6 levels [9, 39, 40]. These discrepancies in the effect of IL-6 concentration after whole-body cryostimulation may result from distinct body compositions of the non-obese and our obese participants. Our study demonstrates that whole-body cryostimulation caused the pro-inflammatory TNFα to decrease without being accompanied by IL-6 concentration increases. This observation contrasts with previous studies [41], which had already reported that IL-6 inhibits TNFα production.

The last published study demonstrated that a single whole-body cryostimulation decreases muscle, core and skin temperatures [42]. The response to coldness exposure may change blood flow through the subcutaneous tissues, attenuating local hypoxic condition in hypertrophic fat cells. According to Ye and Gamble [43], the adipose tissue hypoxia may reflect a compensatory failure in the local vasculature system in response to obesity. Therefore, better blood flow may enhance oxygen supply and limit the production of reactive oxygen species and counteract inflammation. This is in agreement with the study by Miller and co-authors [24], who indicated that cryostimulation may reduce oxidative stress via a significant increase of the level of total antioxidant status, uric acid in plasma and activity of superoxide dismutase in erythrocytes.

The presence of systemic inflammation at baseline was also confirmed by the elevated, still in reference range, amount of white blood cells in the LCF group. The cold exposures did not significantly influence the haematological profile in our obese subjects. The results obtained contrast with the lately published study by Lombardi [44], who noted haematological changes in rugby players following cryostimulation. The discrepancy of the results may be conditioned by the application of a different cryostimulation procedure by Lombardi as well as differences in body composition of the participants between experiments.

Along with the drop of TNFα, we noted a significant, post-cryostimulation increase of the anti-inflammatory cytokine IL-10 in both groups. However, the baseline concentrations of IL-10 were significantly higher in the LCF than the HCF group. These data suggest that elevated concentrations of IL-10 may be a component of an enhanced defensive response to low-grade systemic inflammation. Initially, we hypothesised that exposure to a temperature of −110 °C exerts an anti-inflammatory effect due to increased adiponectin production. Imbelaut and co-authors [28] indicated that a 120-min exposure to coldness (4 °C) was accompanied by a significant increase in adiponectin levels in young, healthy men. Thus, we assumed that exposure to a considerably lower temperature of −110 °C would induce a similar observable effect of increased adiponectin production. Nonetheless, our initial hypothesis was only partially confirmed by the obtained findings. First of all, 10 sessions of coldness exposure did not cause an elevation in adiponectin concentrations. The basic level of adiponectin in the HCF group was significantly higher than in the LCF, yet the recorded values were still lower than concentrations reported for lean individuals: 2,000–3,000 ng mL^−1^[2]. These results are in agreement with previous studies, which noted hypoadiponectinemia in obese individuals [45, 46]. The already mentioned hypoxic condition in excessive fat tissue induces the oxidative stress via activating NADPH, deregulating adiponectin production [47]. Conditions of cryostimulation may have been sufficient to reduce inflammation; however, the applied number of sessions may have been insufficient to improve adiponectin concentration. Lubkowska and co-authors [48] revealed that the beneficial effect of whole-body cryostimulation depends on the number of applied sessions.

Interestingly, we observed a positive correlation between skeletal muscle mass and adiponectin. What is more, the significance of this correlation was strengthened after the series of cryostimulation. It might have resulted from a significant decrease of the pro-inflammatory cytokine TNFα. It has been shown that TNFα suppresses the transcription of the adiponectin gene in adipocyte cells [49]. Moreover, previous studies have revealed that resistance training, known to increase muscle mass, have caused the plasma adiponectin concentration to rise [13].

As it has been consistently reported, obese individuals exhibit elevated levels of leptin [2, 50, 51]; hence, in this study, we attempted to examine the effect of whole-body cryostimulation on this adipokine. The applied protocol of coldness exposures did not affect the concentration of leptin; however, significant differences in leptin concentrations noted between the groups at baseline were sustained throughout the period of coldness treatment. As expected, compared to the LCF, the HCF group exhibited lower concentrations of circulating leptin and higher concentrations of leptin receptor, both within the recommended ranges for lean subjects [52]. Consequently, our data clearly indicate that the maximal oxygen uptake, expressed in mL per kg skeletal muscle mass, correlated negatively with leptin concentrations but positively with leptin receptor levels. These data also suggest that the amount of muscle mass and the metabolic capacity are crucial for regulating leptin levels.

In the LCF group, the elevated leptin levels were also accompanied by higher concentrations of visfatin—the adipocytokine associated with obesity and visceral fat [7, 53]. Visfatin was also elevated in the HCF group. Interestingly, although the HCF group had lower concentrations of visfatin than the LCF group, its visfatin levels before cryostimulation were twofold higher than values recorded for non-obese and trained rowers examined (7.0 ng mL^−1^) by Jurimae [54].

In the current study, at baseline, circulating visfatin levels correlated positively with IL-10 (not shown) and inversely with cardiorespiratory fitness in all participants. This was most likely due to the fact that visfatin may induce the expression of the anti-inflammatory cytokines, IL-10 and IL-1ra [55]. The 10 sessions of cryostimulation triggered changes in the visfatin concentration in both groups, causing an increase in the HCF group but a decrease in the LCF group. Previously, Haus et al. [56] observed a reduction in visfatin concentrations after 12 weeks of aerobic exercise training in older, obese individuals. The data gathered may be explained by the fact that, under cold conditions, shivering in the HCF group was intensified to a greater extent than in the LCF group due to their larger fat tissue content. Shivering can lead to enhanced visfatin synthesis by the skeletal muscles, which was noted to be the source of this cytokine [57, 58]. These speculations might be confirmed by the positive correlation between skeletal muscle mass and visfatin concentration, which appeared after coldness exposure in all participants.

In addition, the changes in resistin concentration showed a similar tendency, such as the above-mentioned visfatin alternation. Resistin concentration, which was also twofold lower at baseline in the HCF group, dropped after exposure to coldness in LCF, whereas in the HCF group it unexpectedly increased. The elevated values at baseline in both groups confirmed previous observations that resistin links obesity and inflammation [59]. However, this adipokine is released mainly in human by macrophages [60, 61], not just adipocytes. Therefore, in this study, correlations between fat mass and resistin were scarcely observed. The decrease of resistin in the LCF group may have partially resulted from the reduction of the pro-inflammatory cytokines, IL-6 and TNFα, which are known to increase reactive oxygen species production [62]. Recently, elevated resistin concentration has been associated with decreased indexes of oxidative stress; thus, it is possible that, in some conditions, its synthesis could be up-regulated in response to reactive oxygen species [63]. However, we have no explanation for why resistin was increased in the HCF group after cryostimulation.

## Conclusions

In conclusion, whole-body cryostimulation application in obese individuals requires further investigation to address the following limitations of this study: a small number of subjects and a limited schedule of blood collection, which should have been adjusted to the work duties of participants. Further investigation should examine how long these induced changes are sustained. To our knowledge, this is the first study to report an anti-inflammatory effect of whole-body cryostimulation on the secretion of cytokines in obese men. Thus, our findings suggest that the designed procedure could be applied in obese individuals, yet its effectiveness also depends on cardiorespiratory fitness. Moreover, this procedure might support dietary modifications in weight loss through changes in adipokine concentration, which is involved in appetite regulation. Therefore, the whole-body cryostimulation can be a supplementary method (due to its low financial cost, it is also increasingly available: 10 exposures = approx 120 Euro) involving exercise, and can possibly be used in some medical procedures and diets aimed to reduce systemic inflammation in obese individuals.
